# Metal Allergy: State-of-the-Art Mechanisms, Biomarkers, Hypersensitivity to Implants

**DOI:** 10.3390/jcm11236971

**Published:** 2022-11-25

**Authors:** Magdalena Zemelka-Wiacek

**Affiliations:** Department of Clinical Immunology, Wroclaw Medical University, 50-367 Wroclaw, Poland; magdalena.zemelka-wiacek@umw.edu.pl

**Keywords:** metal hypersensitivity, metal allergy, allergic contact dermatitis (ACD), patch test, nickel, cobalt, chromium, implants, metal sensitivity

## Abstract

Metal allergy is mainly an environmental disorder which can cause allergic contact dermatitis. Environmental metal exposures include jewelry, everyday metal items, mobile phones, leather, metal-rich food and implants, including stents or anchors. While consumer exposure is liable for the majority of metal hypersensitivity cases, the significance of occupational exposure to metals remains relevant. Although the most common metal allergens are nickel, chromium, and cobalt; however, lately, gold, palladium, titanium, and some others have also attracted attention. This review highlights advances in metal allergy mechanisms, biomarkers for potential patients’ stratification as well as biological treatments. The most recent evidence of human exposure to metal for risk assessment is discussed, as well as the relationship between the occurrence of metal hypersensitivity and implanted devices, including non-characteristic symptoms. The latest data on the diagnosis of metal hypersensitivity are also reported.

## 1. Introduction

The contact dermatitis is a disease driven by repeated contact with contact allergens or irritants, manifested in allergic contact dermatitis (ACD) and/or irritant contact dermatitis [1]. Contact allergens are predominantly low molecular weight chemicals; of which more than 4000 have been recognized. Metal ACD has varying clinical presentations depending on the particular metal. Exposure to metals might be occur in pure forms, plating, or alloy, through the contact with everyday items, metal-rich food, or biomedical devices, including dental implants. Genetic defects affecting structural proteins of the skin barrier may also contribute to an increased risk of ACD [2]. The gold standard of diagnosis–patch tests (PT) require prudent interpretation of delayed or potential false-negative results and irritant reactions [3]. 

**Scheme 1:**
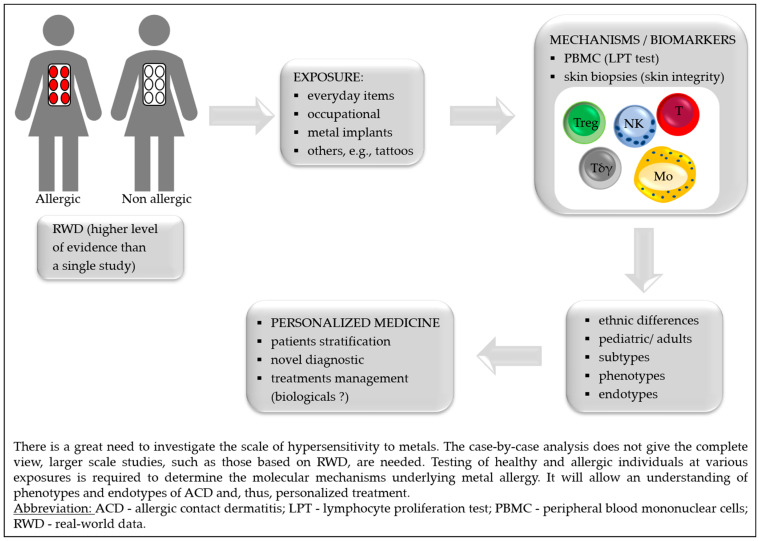
Modern health care of inflammatory skin diseases.

Modern health care is an individualized response to patients’ symptoms, integrating precision diagnosis and personalized treatment. Polyomics, systems biology, and big data have confirmed an intricacy and dynamic variability in allergic disease between individuals and regions. The precision medicine approach stratifies patients based on disease mechanisms to optimize the management of disease together with the health costs [4]. Ethnic differences, extrinsic and intrinsic subtypes, pediatric and adult subtypes, individual clinical manifestations, and finally endotypes which define the molecular mechanisms underlying the visible features/phenotype make up the essential model of the personalized approach to allergy management (Scheme 1) [5]. 

The treatment of inflammatory skin diseases, including ACD, is transforming, emphasizing the need to develop experimental models of skin inflammation to predict treatment responses. A molecular understanding of metal-triggered ACD mechanisms provides an opportunity to develop new drugs for personalized treatment using monoclonal antibodies (mAb) or small molecules targeting the immune response key molecules and cytokines. Other skin-allergy symptom relief solutions are also possible, e.g., nanomaterials can capture metals; mesoporous silica nanoparticles, which are strong nickel (Ni) chelators. It was found to be biocompatible, effective and safe to use for patients with prolonged Ni exposure [6].

This review aims to briefly update the current metal exposures, diagnostics, mechanisms and biomarkers, and the clinical implications of metal implants and biological therapies.

## 2. Exposure

Humans are constantly exposed to metal ions/salts; allergy to metals is the most prevalent contact allergy in developed societies. More information on the oral, cutaneous, and inhalational bioavailability of metals in humans under different dosing regimens and levels is needed for precise risk assessments. Studies with large numbers of sensitized and non-sensitized individuals, different dosing regimens and dose levels are urgently required. 

**Cutaneous and inhalation exposure:** Metal sensitization may cause dermatological disorders. In the Canary Islands, from 1568 patients that underwent PTs, most contact dermatitis patients were older than 40 years, and the main allergen eliciting positive reactions was Ni (36.5%) [7]. Similarly, in the group of 546 patch tested patients, the most common metal allergen in Lithuania was Ni, and women were more often sensitized [8]. In a group of 1919 children, 6% had an allergic reaction to cobalt (Co). ACD triggered by Co should be suspected with dermatitis in a diffused generalized distribution, trunk, or face [9].

Presumably due to their daily exposure to cosmetic products and jewelry, the urine Ni level was significantly higher in females than males on average. Similar occurrence was found in participants who used penetrating jewelry such as earrings and piercings (regardless of gender), compared to subjects not using such jewelry [10].

It was found that potassium dichromate, both in petrolatum and aqua can penetrate the skin, which is important for potential human exposure [11].

Occupational skin diseases appear or exacerbate regarding work and are the second most common type of occupational disease in the world. Nine welding workers had erythematous papules/patches and itching in various areas of the body. The Ni dust was in constant contact with exposed skin, and Ni level exceeded the norm. In two workers, occupational allergic contact dermatitis (OACD) to Ni was confirmed based on a PT [12]. OACD was most frequent in construction workers (45%), the mainly involved area was hands, and the most frequent allergen was chromium (Cr) in cement [13]. It was found that precious metal refinery workers are exposed to non-platinum group metals: lead (Pb), Co, Ni, copper (Cu), arsenic (As) and silver (Ag), with exceeded occupational limits of the South African [14]. The occupational metal ions released at the workplace might entail legal ramifications regarding insurance law.

**Oral exposure:** Ni-rich food is an excellent source of exposure, mainly breakfast cereals, soy products, chocolate spreads and legumes. It demonstrates an evolution in potential risk to human health to Ni exposure due to the shift towards a more plant-based diet [15,16,17,18,19,20]. Ni-allergic contact mucositis (Ni-ACM) is a disorder where Ni-containing food can impact previously sensitized patients and can be diagnosed by a Ni oral mucosa PT (omPT) [21]. Celiac disease patients on a gluten-free diet with positive Ni-omPT displayed a recurrence of gastrointestinal and extraintestinal symptoms, although serological and histological remission has been reached. Relief of symptoms appeared after a gluten-free-low-Ni diet [22]. Irritable bowel syndrome-like disorders are also present in endometriosis. In women with a positive Ni-omTP, a low-Ni diet affected gastrointestinal, extra-intestinal, and gynecological symptoms reduction [23]. An association has also been found between gastroesophageal reflux disease and a low-Ni diet by improving symptoms [24]. Ulcerative colitis (UC) patients often had significant Ni or palladium (Pd) hypersensitivities confirmed by a PT. All subjects had metallic dental implants, implying that exposure to Ni is possible involvement in UC pathogenesis [25]. 

IgE-mediated reactivity to lipid transfer proteins (LTP) is a group of highly conserved proteins mainly found in fruits. They represent the leading cause of primary food allergy in adults in Mediterranean countries. The prevalence of systemic Ni allergy syndrome (SNAS) in the LTP allergic population is clinically relevant [26]. SNAS and Ni-ACD are very common among overweight/obese patients. Ni exposure leads to abnormal production/release of growth hormone (GH). In addition, Ni-allergic patients show GH-insulin-like growth factor 1 (IGF1) axis impairment, probably by increasing the inflammation in the pituitary gland [27].

**Releasing metals from items:** Exposure to metals and their sensitization potential is extremely difficult to assess. The exact composition of the objects we encounter is often unknown, and the composition is not uniform, making diagnosis difficult, e.g., the impact of Ni in tattoo inks is unclear. A positive PT is not sufficient to verify the reaction following tattooing. Epidemiologic case–control studies with regular biopsies of healthy and inflamed tattoos and PTs would facilitate comprehending the role of Ni in tattoo ink allergies [28]. 

Furthermore, the release of metal from objects is not easy to estimate. The gold standard for Ni release assessment is EN1811 test, which has reproducibility limitations [29]. The proposed alternative dimethylglyoxime (DMG) spot test has high specificity but low sensitivity, which undermines its usability, especially when Ni release is low. It was proven that Cu ions could have a masking effect, resulting in an inaccurate reading of the DMG spot test to Ni [30]. The Ni release from everyday products is widespread and often above the DMG test limits. It might be potentially dangerous for Ni-sensitive subjects [31]. Metal parts of laptops and mouses release Ni [32]. Micro-needling made from stainless steel repeatedly puncturing the skin may release Ni [33]. Ni and Co is released in allergology-relevant amounts from beauty tools [34] and metal hairdressers’ tools [35,36]. The European Union established a limit of 0.2 μg/cm^2^/week Ni release for the items by the Directive in 2004 [37]. The excessive Ni release from earrings was found in more than 15% of tested earrings [38,39], also Cr and Co were released [40]. Ni release depends on the solution pH; at pH 4, the release is the greatest, which is essential for the stainless-steel crown in dentistry [41]. 

**Types of metal that can sensitize:** Ni is not the only metal that can sensitize, together with Cr and Co are the most common metals that cause allergic reactions. A patient had itchy erythema confined to the bilateral antihelix. A mono-positive PT revealed gold (Au) hypersensitivity, also the headphones he was using contained Au-plated metal parts. After stopping using them, there was no recurrence of symptoms [42]. Iron (Fe) can be a relevant sensitizer, especially in complicated knee arthroscopy [43]. Aluminum (Al) salts are adjuvants found in many vaccines. Although rare, patients have reported cutaneous reactions, including ongoing pruritic subcutaneous nodules at the injection site. In most cases, delayed reactions are not contraindications to further vaccine administration. However, it should be evaluated case-by-case basis offering alternative Al-free vaccines [44,45]. Allergy from contact exposure to Al, e.g., topical medicaments and deodorants might be more common than thought (7 case reports) [46,47]. Sunscreens containing Al might lead to ACD in pediatric patients [48]. Unfortunately, Al-PT is only positive when there is a strong Al-allergy [49]. 

## 3. Diagnostics

**Patch tests (PTs):** PTs are the gold standard for the diagnosis of allergic hypersensitivity [50]. It is known that PT readings on day seven (D7) may show additional positive reactions. Metal allergens and older age were predictive for late positive reactions. Within the tested allergens, without D7 readings, on average, 12% of sensitizations would have been missed [51,52]. Long-lasting allergic PT reactions (LLAPTR) are positive PT reactions lasting more than two weeks. A 90-year-old dental patient had a positive PT on D2 and D4 for platinum (Pt), Pd, and indium (In), but for Au the test was negative until D45 [53].

Ideally, the PTs should be performed while the patient is not taking biologics. Unfortunately, this is not always possible, so clinicians need to assess the risks and benefits of testing during therapy [54]. The precision of PTs after dupilumab is under consideration. Reactions may stay positive (with no dampening effect), change to negative (false-negative reactions), or become newly positive after its administration, indicating that patient-specific factors should be taken into consideration [55,56,57]. Secukinumab (anti-IL-17 mAb) showed no significant reduction in Ni-PT in patients with ACD (with confirmed Ni allergy before) [58]. 

When hypersensitivity to metal implant is suspected, the PT should be done for confirmation. Additionally, the identification of implant compositions should be accomplished before potential removal. The patient underwent a skin grafting that was covered with a tattoo. An allergic reaction to anchors or tattoo inks was suspected. The DMG spot test result was negative for metal devices. Finally, an inductively coupled plasma-mass spectrometry analysis revealed the release of Ni from the anchor [59].

Ag is widely used topically because of its anti-microbial properties. It should be considered to include Ag as an extension of PTs of dermatitis in subjects with skin ulcers since it is not present in some of the commercial series. Hereby, a greater number of cases of ACD to Ag could be identified earlier [60].

Ni allergen is present in different PT lines, e.g., ICDRG baseline, European or Swedish baseline, but it occurs as Ni sulfate 200 μg/cm^2^ in TRUE Test, or Ni sulfate 2.5% petrolatum (pet.) and 5% pet. The reactions vary, and most positive reactions were found in 5% pet., TRUE Test and 2.5% pet. shown similar responses [61].

**In vitro tests:** The in vitro lymphocyte proliferation test (LPT) can be an additional method in case the skin is not the primary organ of exposure. The classical LPT uses tritiated thymidine, so a radioactive-free LPT test with carboxyfluorescein succinimidyl ester (CFSE) was proposed, which furthermore can distinguish T cell subsets and assess the released cytokines [62,63]. However, the read-out method can affect the sensitivity of the LPT; it was confirmed that ELISA or flow cytometry provides the best detection of sensitization in the context of allergies [64]. In the culture of PBMC performed by LPT, the metal-reactive (Cr, Ni, and Co) T helper lymphocytes (Th cells) with high CD45RO expression and co-expression of cutaneous lymphocyte-associated antigen (CLA) and C-C Motif Chemokine Receptor 6 (CCR6) were identified. Th cells identified individuals with a positive Ni-PT with 100% sensitivity and 92% specificity [65].

**Other tests:** Urine Ni concentration is an effective predictor of Ni exposure but not of an allergy. The urine Ni concentrations were not statistically different in allergic patients with Ni-positive PT compared to negative controls. However, the urine level of Ni correlated with lifestyle [10]. 

## 4. Mechanisms and Biomarkers

The discovery of the molecular mechanisms by which contact allergens cause skin sensitization has a potential implication for treatment decisions. The biomarkers might facilitate the diagnosis of metal hypersensitivities and enable patient stratification for potential treatment strategies [66]. Key advances in the understanding of metal allergy mechanisms and biomarkers are presented in Table 1.

Peripheral blood mononuclear cells (PBMC) from allergic and non-allergic donors stimulated in vitro with Ni, Co, or Pd salts showed an increased frequency of metal-specific-CD154^+^ (CD40L) CD4^+^ memory T lymphocyte (Tmem) cells. Overrepresentation of specific individual gene segments, including α-chain V-segment (TRAV9-2) and complementarity determining region 3 (CDR3) histidine, defines different T cell receptor (TCR) repertoire features that underlie metal ion binding and cross-reactivity [67,68]. Ni-induced contact allergy is represented by a broad cytokine response with cytokines released from type 1, type 2, type 9, type 17, and type 22 cells. Type 2 cytokines showed the strongest correlation, and interleukin (IL)-5 remains potentially the strongest biomarker for Ni allergy [69]. 

The PT results might doubt the clinical symptoms because of cross-reactivity between different metals. In a murine model of ACD, the sensitization was performed with Ni or Cr, but the challenge was performed with Pd. The Pd-cross reactive allergy was noticed, and the mucosal-associated invariant T and invariant natural killer T (iNKT) cells were involved [70]. The LPT test and IL-5 production assessment distinguished between Pd allergy and cross-reactivity with Ni [71]. Ni strongly positive PT was disclosed in a patient with papular lesions on the buccal mucosa. However, the alloy prostheses in the oral cavity did not contain Ni but Pd. The lesions had healed entirely three months after the prostheses were removed without any other treatment [72].

Healthy subjects had a topical Ni administration, and then the biopsies were collected. Th17 (IL-17A), Th1 (C-X-C motif chemokine ligand 10 (CXCL10)), and Th2 (IL-4 receptor (IL-4R)) immune responses were noticed. CD3^+^ T-cells, CD11c^+^ myeloid dendritic cells (DC), DC lysosomal associated membrane glycoprotein (DC-LAMP)^+^ mature DCs, myelin basic protein (MBP)^+^ eosinophils and forkhead box P3 (FOXP3)^+^ T regulatory cells (Tregs) were significantly enriched. Ni application caused barrier defects by reduction of terminal differentiation (filaggrin (FLG), FLG2, loricrin (LOR), and late cornified envelope proteins (LCEs)), tight junction (claudin (CLDN)1/CLDN8), and lipid metabolism (fatty acid 2-hydroxylase (FA2H), fatty acid binding protein 7, brain (FABP7)) AD-related markers [73]. After positive Ni-PT, skin biopsies were taken at different time points (0 to 96 h). Major significant changes were a higher rate of proinflammatory macrophages (M1), activation of neutrophils, natural killer (NK), CD4^+^ Tmem, CD8^+^ T and mast cells. Matrix metallopeptidase 12 (MMP12) in M1 cells and suppressor of cytokine signaling 3 (OCS3) were upregulated. Gene expression profiles were analyzed, and NK cell infiltration and cytotoxic pathways were found. Simultaneously the frequency of anti-inflammatory macrophages (M2), resting mast cells, T gamma-delta (Tγδ) cells, and Treg cells decreased [74].

**Table 1:** Key advances in metal allergy mechanisms and biomarkers.

Outcomes	Exposition to Metal	Ref.
↑ metal-specific CD154^+^ CD4^+^ Tmem (overexpression of TRAV9-2 and CDR3 histidine)	Allergic and non-allergic subjects stimulated with Ni, Co or Pd (PBMC)	[67,68]
↑ IL-5	Ni-allergic patients (PBMC)	[69]
	Ni-allergy patients, differentiation between independent Ni or cross-reactivity of Ni/Pd allergy (PBMC)	[71]
Cross reactivity between Ni/Cr and Pd	Sensitization with Ni or Cr, challenge with Pd (mice model)	[70]
Skin barrier defects: ↓ terminal differentiation—FLG, FLG2, LOR, LCEs, tight junction—CLDN1/CLDN8, lipid metabolism—FA2H, FABP7	Biopsies from healthy subjects after Ni-topical application	[73]
Cellular infiltrates: ↑ CD3^+^ T, CD11c^+^ myeloid DC, DC-LAMP^+^ mature DC, MBP^+^ eosinophils, FOXP3^+^ Treg		
↑ M1, mast cells, neutrophils, NK, CD4^+^ Tmem, CD8^+^ T	Biopsies from Ni-allergic patients	[74]
↓ M2, resting mast cells, Tγδ, Treg		
↓ SBSN	Ni-allergic patients (serum)	[75]
↑ Sema3A (activates MAPK and TNF-α)	Ni-induced allergy (mouse ear tissue)	[76]
↑ TSLP in keratinocytes and TNF-α in epithelium	OLPs metal-allergy patients	[77]
↑ IL-6, CXCL8, CCL2, CCL5, and CCL20	RHS exposure to Ni and *Streptococcus mitis* exposure	[78]
Lipid profile: ↑ cholesterol, DAG, MAG	Non-allergic skin exposed to Co	[79]
Lipid profile: ↑ DAG	Non-allergic skin exposed to Cr	
Lipid profile: ↓ DAG and MAG	Non-allergic skin exposed to Ni	
LC emigration of the epidermis (in an IL-10 but not IL-1B dependent way)	RHS exposure to Ti	[80]

Abbreviations: ↑ = increase; ↓ = decrease; + = positive; CCL = C-C motif ligand; CD = cluster of differentiation; CDR3 = complementarity determining region 3; CLDN = claudin; Co = cobalt; Cr = chromium; CXCL = C-X-C motif chemokine ligand; DC = dendritic cell; FA2H = fatty acid 2-hydroxylase; FABP7 = fatty acid binding protein 7, brain; FLG = filaggrin; FOXP3 = forkhead box P3; IL = interleukin; LAMP = lysosomal associated membrane glycoprotein; LCEs = late cornified envelope proteins; LOR = loricrin; M1/M2 = proinflammatory/anti-inflammatory macrophages; MAG/DAG = mono/diacylglycerols; MAPK = mitogen-activated protein kinases; MBP = myelin basic protein; Ni = nickel; NK = natural killer cells; OLP = oral lichen planus; PBMC = peripheral blood mononuclear cells; Pd = palladium; RHS = reconstructed human skin; SBNS = suprabasin; Sema3A = semaphorin; Tmem = memory lymphocytes T; TNF-α = tumor necrosis factor-alpha; TRAV9-2 = α-chain V-segment; TSLP = thymic stromal lymphopoietin; Tγδ = T gamma-delta cells.

Suprabasin (SBSN) is a differentiation-associated protein of the epidermis that is highly expressed in the upper layers of stratified squamous epithelium in various organs, including the skin and upper digestive tract. A decrease in SBSN is observed in Ni allergy patients compared with subjects without metal hypersensitivity. Ni is mainly absorbed from the upper digestive tract, so Ni-allergic patients may have reduced SBSN expression in the upper gastrointestinal tract, which can promote Ni absorption, enhancing allergy. The level of SBSN is lower in intrinsic atopic dermatitis patients, which have a high complication rate of Ni-allergy than in extrinsic patients [75].

Semaphorin 3A (Sema3A) is an effective immune cell migration and regulation modulator. Its expression increased in Ni allergy-induced mouse ear tissue and keratinocytes. The deletion of SEMA3A reduced edema and ear swelling. Sema3A boosts metal allergy by activation of mitogen-activated protein kinases (MAPK) and tumor necrosis factor-alpha (TNF-α) production, and should be explored as a potential target for preventing and treating metal allergy [76]. Metal allergy is linked to enriched expressions of thymic stromal lymphopoietin (TSLP) in keratinocytes and augmented TNF-α levels in the epithelium. The expansion of T cells at the lesion site collaborated with the formation of the oral lichen planus (OLP). T cells infiltration via TSLP-TSLP-receptor (TSLPR) signaling, and TNF-α production was higher in OLPs in metal-allergy patients compared to healthy controls [77].

Skin and oral mucosa host abundant microbes and might also be exposed to metals, and the commensals may impact the organism’s response to metals. Ni-mediated cytokine secretion IL-6, CXCL8, C-C motif ligand (CCL2), CCL5, and CCL20 is enriched by *Streptococcus mitis* in reconstructed human skin (RHS) but not in the gingiva (RHG), while Ti suppresses cytokines induced by *S. mitis* in RHS. Moreover, the co-application of metal and *S. mitis* differentially regulate Toll-like receptors (TLR)1 and 4 expressions [78].

The lipid profile in the stratum corneum and viable epidermis of the skin altered upon topical exposure to metals metal (Co, Cr, and Ni) in a healthy donor. The analysis revealed that Co increases cholesterol, and Co and Cr increase diacylglycerols (DAG) levels in the stratum corneum and epidermis. Co also raised the monoacylglycerols (MAG) level in the epidermis. In contrast, Ni reduced DAG and MAG in the epidermis [79].

Titanium (Ti) salts were shown to be irritants rather than sensitizers. RHS model containing primary differentiated keratinocytes and CD1a^+^-Langerhans cells (LCs) were topically exposed to Ti salts. Although LC migrated out of the epidermis, which was CCL5 mediated, with an increased IL-10 messenger RNA (mRNA), but not IL-1B or CCR7. This could suggest that implants which were containing Ti caused inflammation and cytotoxicity due to localized irritation and corrosion rather than metal hypersensitivity [80]. This is confirmed by the studies where Ti hypersensitivity in dental implants was linked with innate immunity induced by hyperreactivity macrophages to Ti particles [81]. 

Systemic retinoids cause can damage the skin barrier, which is assumed to augment the onset of ACD. A non-atopic patient had systemic isotretinoin therapy because of acne vulgaris. After two months, eczema occurred in places where he was wearing jewelry. Previously he had no lesions in these areas. The PT was positive (+++) for Ni [82].

Interestingly, smell and taste are altered in Ti and Ni-hypersensitive orthodontic patients. The mechanism of this reaction is unknown [83]. 

## 5. Implants

Metal sensitivity may be a rare cause of prosthetic joint failure or loosening. An implant allergy is diagnosed by a PT, and implant removal is crucial to both diagnosis confirmation and treatment. The revisions are not such a problem because hypoallergenic implants may relieve symptoms. In 2013, an outline was presented for diagnosing metal hypersensitivity caused by an implant: confirmation of eruption overlying the implant (chronic dermatitis can begin weeks to months after implant use), diagnosis by positive PT, and symptoms resolution after the implant removal [84]. However, to date, there are no guidelines in this regard. The latest evidence suggests that there is a link between (implants) metal, in particular Ni allergy, and autoimmunity that may lead to similar clinical outcomes. An explanation of their potential mechanisms will support a more successful and safer treatment [85]. Advances on hypersensitivity to medical implants, including unusual symptoms are presented in Table 2.

**Joint arthroscopy:** 1354 total knee arthroscopy (TKA) cases were analyzed with the following conclusions: patients undergoing TKA with a history of metal hypersensitivity seem more likely to encounter adverse outcomes than patients with no history of metal allergy [86]. In 233 patients that underwent TKA and had no prior metal hypersensitivity diagnosed, 15.8% had positive patch test (mainly to Cr, Ni, Co), and 12% were symptomatic for pain and subsequent loss of implant function [87]. Patients with a known metal-allergy history were applied to standard or coated knee implants. No differences in knee function, but an increase in plasma Cr level in the standard group was found [88]. Self-reported metal allergy is used as a screening tool for implant selection. In 168 patients that reported having metal allergy, no differences were found in early functional implant outcomes after TKA in groups receiving Ni-free or Co-Cr-Ni implants [89]. Another study confirmed, that no local or systemic symptoms of hypersensitivity to Co-Cr implant was found in patient who reported self-metal allergy [90]. Hypoallergenic implants were used in TKA patients with a history of metal allergy with a ten-year survivorship up to 95% [91]. 

Patients were reported where it was determined that metal allergy to orthopedic implants led to surgery-related complications. The most common symptoms for patients with metal (Ni) hypersensitivity-associated failures are soft tissue reactions, including delayed wound healing and/or recurrent wound issues. Hypersensitivity symptoms closely mimic infection and implant failure, therefore diagnosis is challenging [92,93].

At least one metal hypersensitivity incidence in patients enrolled for TKA with no previous clinical symptoms was only in 3% subjects [94]. Regardless, in another study, the preoperative metal PT (MPT) was performed in 16 patients out of 1087 that underwent a total arthroplasty of joint, and the loosening of implant occurred in 0.3%, none of whom had a metal allergy history or underwent the MPT [95].

It was found that PTs may be beneficial in patients with a metal hypersensitivity history. The predictive value was significantly high when the test results were strongly positive, which correlated with clinical outcomes of implant allergy [96]. Twenty-nine patients before hip, knee, or shoulder arthroplasty had a PT with ten metals performed. Four positive reactions were observed to Ni and Cr. Six months after the surgery, a PT was performed again, and the contact hypersensitivity increased from a baseline by 13.8%. Half of the patients, who received an implant with Ni, developed sensitivity. However, two patients developed eczematous lesions around the implant, although the PTs were negative twice [97]. 

**Revision of arthroscopy**: There are many cases where the resolution of symptoms due to metal allergy was achieved by hypoallergenic revision of TKA [98]. 39 patients after TKA with pain in the knee and positive PT to metal, improved pain scores, walking function, and range of motion following revision to a hypoallergenic implant [99]. No significant differences in symptoms of revision surgery were found between patients with positive versus negative metal-PT [100]. LTT results alone are insufficient for diagnosing TKA failure due to a metal allergy. There was no correlation between histopathologic assessment and the LTT test [101].

**Endovascular implants:** ACD is more often identified in patients with endovascular implants. The ACD can conduct in-stent restenosis or prominent eczematous reaction overlying the implant and the need for removal. Ni allergy is linked with a raised risk of adverse outcomes following endovascular device implantation, which was a systematic review of a group of 1740 patients in 78 different studies [102].

**(Metal) dental allergies:** An oral lichenoid contact lesion (OLCLs) may be caused by allergic contact stomatitis—a hypersensitivity response to dental materials, including amalgam. 30 patients with OLCLs showed complete or partial improvement after the removal of dental metal. The PT confirmed the hypersensitivity, the most common positive test was for Au and Pd [103]. Of the 359 dental patients who underwent the PT, 241 showed a positive reaction to metal (the most common was Ni), and half of them had metals in their dental prostheses, because of the symptoms and test results, they were diagnosed with a dental metal allergy. In addition, there was a significant correlation between palmoplantar pustulosis (PPP) and dental metal allergy [104]. 

Dental allergies to Ti are rare. In the process of tribocorrosion, Ti particles are released from the implant surfaces, which may cause bone loss because of inflammation. Ti hypersensitivity to dental implants was confirmed in 25 studies [105]. Still, the Ti allergy remains controversial, especially the recent studies shown suggested that it is rather irritant than sensitizing [80].

**Table 2:** Advances on hypersensitivity to medical implants, including unusual symptoms.

Metal that Caused Hypersensitivity/Implant Type	Symptoms	Ref.
Multiple metals/orthopedic implants: hip, knee shoulder joint	Different; the most common—delayed wound healing and/or recurrent wound issues after the implantation, joint failure or loosening	[86,92,93,98,106]
Ni/endovascular implants (stents)	In-stent restenosis or prominent eczematous reaction overlying the endovascular implant, eosinophilia	[102,107]
Co, Ni/drug-eluting stents	Pruritic rash with hypereosinophilia	[108]
Au and Pd/dental implant	Oral lichenoid contact lesion (OLCLs)	[103]
Ti/dental implants	Rash, urticaria, pruritus, redness, dermatitis and facial eczema, pain, hyperaemia of soft tissues, swelling in submental and labial sulcus, gingival hyperplasia acne-like facial inflammation	[105]
Ni/metal anchors	Erythematous and vesicular lesions around the grafted tattoo skin, but the tattoo was not affected (placed with clips or anchors)	[59]
Ni/stainless-steel skull pins	Erythema on sites of the head where the skull pins inserted	[109]
Ti/cervical implant	Persistent refractory neck pain; subsequently, after eight years, a planter rush	[110]
Ti/metal clips for cholecystectomy	Right upper quadrant pain, diarrhea, and nausea	[111]
	Low-grade fever, nausea, vomiting, joint pain, bloody diarrhea	[112]
Co, Ni, Hg/metal clips for cholecystectomy	Myalgia, joint pain and tenderness, mental fogginess, mild forgetfulness, irritable bowel syndrome, stomach cramps, dry skin and hair, hair loss	[113]
Cu/intrauterine device	Cutaneous eruption	[114]
Multiple-metals/dental implant	Palmoplantar pustulosis (PPP) and periodontitis	[104,115]
Au/dental implants	Oral lesions with characteristic Wickham’s striae	[116]
Ti/temporary tissue expander	Well-demarcated, erythematous plaque over the left breast reconstructive breast surgery	[117]

Abbreviations: Au = gold; Co = cobalt; Cu = copper, Hg = mercury; Ni = nickel; Pd = palladium; Ti = titanium.

**Case studies:** In a hairdresser, recalcitrant lateral epicondylitis was covered with the donor tattooed skin and placed with metal clips and anchors. Six weeks later, the patient reported the erythematous and vesicular lesions around the grafted skin, but the tattoo was not affected. A positive Ni (+++) PT was revealed. An allergic reaction to ink, clips or anchors was suspected, however, finally the symptoms resolved after the anchors’ removal. The delay between the surgical procedure and the occurrence of the skin lesions can be longer than in traditional ACD, even weeks or months can be required for the allergen release making diagnosis difficult [59]. Further, a CD to stainless-steel skull pins, containing, among others, Ni, was observed in patient with no allergy history after nine days post-surgery [109].

Symptoms of metal allergy might not be characteristic of hypersensitivity reactions such as erythema, itchiness, blistering, and exudate. After cervical Ti alloy implant surgery, a woman had persistent refractory neck pain; subsequently, after eight years, a planter rash developed. She was diagnosed with the metal hypersensitivity, and also with PPP, because of rash on her foot. After the implant was removed, the symptoms of pain and rash improved [110]. A patient had right upper quadrant pain, diarrhea, and nausea after laparoscopic cholecystectomy. Although numerous diagnostic tests were not unequivocal, the patient lived with pain for five years. After the surgical removal of Ti clips, the symptoms improved during the week postoperative. The PTs were not done before surgery, despite a history of Ni allergy [111]. This is in line with other cases where patients had a confirmed allergy to metal (Ni, Co, and Ti), and the abdominal pain, fatigue, lethargy, joint aches, and nausea symptoms after cholecystectomy were relieved after surgical clips removal [112,113]. 

A woman started to have a recurrent cutaneous eruption three months after intrauterine device (IUD) insertion. Systemic allergic dermatitis (SAD) to Cu was confirmed. Cu-containing IUDs increase medical concerns as they are planned to be worn for many years [114].

A patient was diagnosed with systemic metal allergy after a dental prosthesis, and subsequently, a PPP was developed. Therefore, for the aortic valve replacement an Avalus bioprosthetic valve was chosen which does not contain metal in the stent [115]. 

A patient with a history of atopic disease developed oral lesions with characteristic Wickham’s striae. The performed broad PT panel was mono-positive for Au. The oral lichen planus resulted from the Au dental implants [116]. 

A female had a well-demarcated, erythematous plaque over the left breast 13 months after reconstructive breast surgery with the placement of a temporary tissue expander (TTE). The PT was positive for a few metals, including Ti, and the port of the TTE contained Ti. Dermatitis resolved after the tissue expander disposal [117].

## 6. Treatment with Biologicals

In ACD corticosteroids treatment remains the first choice of therapy. However, the chronic character of the disease and many side effects related to corticosteroids continue to be major barriers to their long-term use. ACD has been considered to be T1-mediated inflammation, however response to anti-T2 biologics indicates a pathological role for T2 cytokines. Biologic therapies, namely mAb, which target specific mediators or pathways of inflammation, may be an alternative for treating resistant cases of ACD [118]. However, there are few reports in the literature on the use of biological treatments, and they should be used with great caution.

A patient developed an intensely pruritic rash with hypereosinophilia after coronary angioplasty with drug-eluting stents (DES) placement. The DES had a nonexposed Pt-iridium (Ir) core with a Co-Ni-Cr-molybdenum (Mo) alloy shell. The PT was positive for Co (++) and Ni (++). Although SAD was suspected, stent removal was not feasible due to requiring coronary artery bypass surgery. Mepolizumab, an anti-IL-5 mAb (IL-5 is crucial for eosinophils development) was administrated with an acceptable result [108]. Another patient with SAD and eosinophilia due to a Ni-containing responded to dupilumab, a mAb that blocks IL-4/IL-13 signaling cascade [107]. 

A patient with Ni-atopic occupational chronic contact dermatitis failed to execute remission under various systemic modalities. Dupilumab treatment for six months resulted in complete remission for the first time in the last five years [119]. A patient with severe Cr-induced ACD with multiple scaly, erythematous plaques scattered over the skin of his hands, head, and neck was successfully treated with dupilumab because of refractory to topical corticosteroids [120]. 

A patient had fatigue and a diffuse erythematous with a pruritic rash after TKA. PTs showed an allergy to Co, Ni, and Au. The treatment with omalizumab, an anti-immunoglobulin E (IgE) mAb, showed just an initial response and after five months, the recurrence of the symptoms. She was then successfully treated with dupilumab [106]. 

## 7. Conclusions

Metal hypersensitivity is becoming a public concern. ACD and SAD caused by metals appear more commonly than previously thought. There are many reports (mainly on a case base) that various metals are released in amounts sufficient to cause a reaction. Ni is the most often tested metal because it is the one that causes allergies most frequently. There is not sufficient data about other metals.

Metal hypersensitivity to implanted devices remains difficult to identify. In scientific evidence, whether a metal implant can cause an allergic or irritant reaction is still debatable. Knowing the full composition of the medical device would help guide further management. Physicians should consider performing diagnostic PTs before implant surgery in patients who report metal/jewelry allergies.

Real-world data (RWD) is needed to recognize the scale of metal hypersensitivity, agreed-on diagnostic criteria and procedures. Based on a meta-analysis, these findings provide a higher level of evidence than a single study that does not consider individual patient data. Novel diagnostic approaches and evidence-based treatment guidelines that consider the practical implications of using phenotyping and endotyping in facilitating the stratification of patients into responding and non-responding populations and defining the theratypes are necessary. More effective treatments with satisfactory safety profiles should be prioritized.
